# Effect of massive blood transfusion on late outcomes after surgical repair of acute type A aortic dissection

**DOI:** 10.1097/MD.0000000000017816

**Published:** 2019-11-11

**Authors:** Fang-Ting Chen, An-Hsun Chou, Victor Chien-Chia Wu, Chia-Hung Yang, Pao-Hsien Chu, Pei-Chi Ting, Shao-Wei Chen

**Affiliations:** a Department of Anesthesiology, Chang Gung Memorial Hospital, Linkou Medical Center, Chang Gung University, Taoyuan City; b Department of Medicine, Chang Gung University, Linkou,Taipei; c Department of Anesthesiology, Xiamen Chang Gung Hospital, Taoyuan; d Department of Cardiology, Chang Gung Memorial Hospital, Keelung Branch and Linkou Medical Center, Taoyuan City; e Division of Thoracic and Cardiovascular Surgery, Department of Surgery, Chang Gung Memorial Hospital, Linkou Medical Center; f Graduate Institute of Clinical Medical Sciences, College of Medicine, Chang Gung University, Taoyuan City, Taiwan.

**Keywords:** blood transfusion, chronic kidney disease, late outcome, mortality, respiratory failure, type A aortic dissection

## Abstract

Massive blood transfusion (MBT) increased mortality and morbidity after cardiac surgery. However, a mid-term follow-up study on repair surgery of acute type A aortic dissection (AAAD) with MBT was lacking. This study aimed to assess the impact of perioperative MBT on late outcomes of surgical repair for AAAD.

There were 3209 adult patients firstly received repair surgery for AAAD between 2005 and 2013, were identified using Taiwan National Health Insurance Research Database. Primary interest variable was MBT, defined as transfused red blood cell (RBC) ≥10 units.

The outcomes contained in-hospital mortality, surgical-related complications, all-cause mortality, respiratory failure, and chronic kidney disease (CKD) during follow-up period. Higher in-hospital mortality (37.7% vs 11.6%; odds ratio, 4.00; 95% confidence interval [CI], 3.30–4.85), all-cause mortality (26.1% vs 13.0%; hazard ratio [HR], 1.66; 95% CI, 1.36–2.04), and perioperative complications were noted in the MBT group. A subdistribution hazard model revealed higher cumulative incidence of CKD (13.9% vs 6.5%; HR, 1.95; 95% CI, 1.47–2.60) and respiratory failure (7.1% vs 2.7%; HR, 2.34; 95% CI, 1.52–3.61) for the MBT cohort. A dose-dependent relationship between amount of transfused RBC (classified as tertiles) and cumulative incidence of all-cause mortality, incident CKD, and respiratory failure was found (*P* of trend test <.001).

Patients with MBT had worse late outcomes following surgical repair of AAAD. The cumulative incidence of all-cause mortality, incident CKD, and respiratory failure increased with the amount of transfused RBC in a dose-dependent manner.

## 1 Introduction

Blood transfusions are frequently performed during cardiac surgeries.^[1]^ Previous studies have indicated that massive blood transfusions (MBTs) lead to immediate and long-term negative complications in major surgeries, including cardiac surgeries.^[2,3]^ These adverse consequences include dilutional coagulopathy, electrolyte imbalance, acid–base abnormalities, hypothermia, transfusion-related acute lung injury, postoperative infection, prolonged ventilator use, longer hospital stay, acute kidney injury, and immunosuppressed events.^[2,4]^ In addition, blood transfusions reduce not only short but also long-term survival rates following cardiac surgeries.^[5,6]^

Acute type A aortic dissection (AAAD) is a life-threatening disease and requires prompt surgical intervention, but is complicated by a high surgical and in-hospital mortality rate, 18% to 31%.^[7]^ Blood transfusion is inevitable in surgical repair of AAAD due to the nature of aortic surgery and dysregulation of the coagulation system.^[8,9]^ Compared to other types of cardiac surgeries, the surgical repair of AAAD results in more serious bleeding, owing to the difficult surgical technique, and disseminated intravascular coagulation-like coagulopathy resulting from pathophysiologic activation of the hemostatic system, and amplified by the use of cardiopulmonary bypass and systemic hypothermia for brain protection.^[8–10]^ Therefore, excessive refractory blood loss is commonly noted in surgical repair of AAAD and requires MBT during the perioperative period. Furthermore, no significant difference was proved in the prevalence of AAAD and bleeding tendency between West and East-Asia populations.^[11,12]^

However, the relationship between the amount of transfused blood products in surgical repair of AAAD and postoperative long-term outcome has never been precisely investigated. Hence, the aim of the current study was to analyze the impact of MBT on long-term outcomes in patients undergoing surgical repair of AAAD by utilizing the national database in Taiwan.

## 2 Materials and methods

### 2.1 Data source

This retrospective, nationwide, population-based cohort study was performed using the Taiwan National Health Insurance Research Database (NHIRD). NHIRD was derived from the single-payer National Health Insurance (NHI) program, which was launched on 1st March 1995, and covered nearly 99% of the 23 million residents of Taiwan. Payments for laboratory tests, examinations, medications, interventions, surgeries, and admissions were audited via Taiwan's Bureau of NHI. Lifesaving cardiovascular surgeries were also reimbursed according to associated provisions. In addition, blood transfusions were also reimbursed if indicated, and the amounts were precisely recorded in the database. The NHIRD did not contain the actual timing for blood transfusion; therefore, we extracted the blood transfusion during the first 30 days of the index hospitalization. Patients with in-hospitalization mortality can be defined as either the patent expired during the index hospitalization, acute terminal stage when discharge, and discharged against medical advice, which mostly happens in Taiwan when there is no more effective way to sustain life and the patient and/or the family wishes to bring patient home for comfort. These data can be extracted from NHIRD; however, the accuracy of data have been validated in previous studies.^[13–15]^ The NHIRD provides a good platform to perform research on the long-term follow-up of severe disease and major surgeries.

Investigators who want to utilize the NHIRD for research purposes are required to follow related laws and regulations, such as the Computer-Processed Personal Data Protection Law and the regulations of associated NHI administrations. We complied with these regulations and obtained documented agreement from relevant supervisors before execution of this study. Diagnoses of diseases in NHIRD are defined based on the International Classification of Diseases, 9th Revision, Clinical Modification (ICD-9-CM). This study was evaluated and approved by the Ethics Institutional Review Board of Chang Gung Memorial Hospital.

### 2.2 Study population

**Figure 1:**
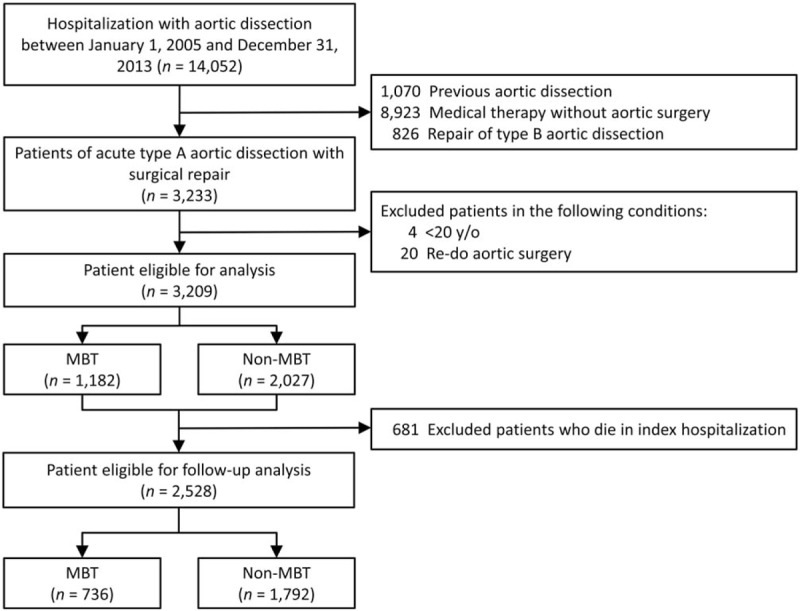
Flow chart of identification and enrollment of the study population. MBT = massive blood transfusion.

This observational, longitudinal, nationwide, population-based cohort study was designed to assess the mid-term clinical outcome of surgical repair for AAAD with varying amounts of perioperative red blood cell (RBC) transfusion. We screened the NHIRD hospitalization records for all patients who received aortic dissection surgery between January 1, 2005 and December 31, 2013. Patients diagnosed with aortic dissection were firstly extracted using ICD-9-CM code 441.0x. We excluded patients who had a diagnosis of aortic dissection between 1997 and 2004. Furthermore, Taiwan NHI procedure codes (billing codes) were applied to recognize patients who underwent AAAD repair surgery or type B aortic dissection repair. We excluded patients with previous aortic dissection, under medical therapy without aortic surgery and those who received type B dissection repair. In total, 3233 patients who received surgical repair for AAAD were identified during the study period. Exclusion criteria applied to this identified sample included: patients aged <20 years, and patients who had undergone repeat aortic surgery. From this, there were 3209 patients eligible for subsequent analysis. In addition, we exclude patients who died during index hospitalization for further analysis follow-up outcomes (Fig. 1). The definition of MBT in this article was: transfused ≥10 units of RBC blood product.^[3]^

### 2.3 Covariates and outcomes

The comorbidities, surgical details, perioperative complications, and late outcomes of the patients were identified by ICD-9-CM diagnosis or Taiwan NHI reimbursement codes. The comorbidity was detected using any inpatient diagnosis before the index hospitalization which can be tracked to year 1997. Surgical details were extracted using Taiwan NHI reimbursement codes, including extension of aortic surgery, additional surgeries during the index hospitalization, and previous cardiac surgery. perioperative complications were detected by either ICD-9-CM diagnosis codes (ie, respiratory failure, new onset ischemic stroke, new onset hemorrhagic stroke, and sepsis) or Taiwan NHI reimbursement codes (ie, cardiogenic shock, re-exploration for bleeding, de novo dialysis, blood transfusion, and intensive care unit duration). All-cause mortality was defined by a withdrawal from the NHI.^[16]^ In-hospital mortality was defined as a withdrawal from the NHI within 7 days of the discharge of the index hospitalization. Late outcomes included newly onset chronic kidney disease (CKD) and respiratory failure. Incident CKD was defined as anyone inpatient diagnosis after the discharge of the index hospitalization. Respiratory failure was verified with an approval of possessing catastrophic illness certificate card. All patients were followed since the index discharge till December 31, 2013 or the date of death, whichever came first.

### 2.4 Statistical analysis

The clinical and surgical characteristics of patients with and without MBT (the study groups) were compared using chi-square tests for categorical variables or *t* tests for continuous variables. Operation-related complications and outcomes were compared between the 2 study groups using logistic regression analysis for categorical outcomes and linear regression for continuous outcomes. Time to event outcome related to death was analyzed using a Cox proportional hazard model. As with other time to event outcomes (ie, CKD or respiratory failure), death during the follow-up was considered as a competing risk and the cumulative incidence was estimated under a subdistribution hazard model. We treated the study groups as independent variables and adjusted for characteristics listed in Table 1 in all regression models. In addition, in order to investigate the potential risk factors for MBT, we introduced covariates which were significant in the prior univariate analyses into a multivariable logistic model with backward selection. *P* value <.05 was considered statistically significant. Data analysis was conducted using SAS version 9.4 (SAS Institute, Cary, NC).

**Table 1:**
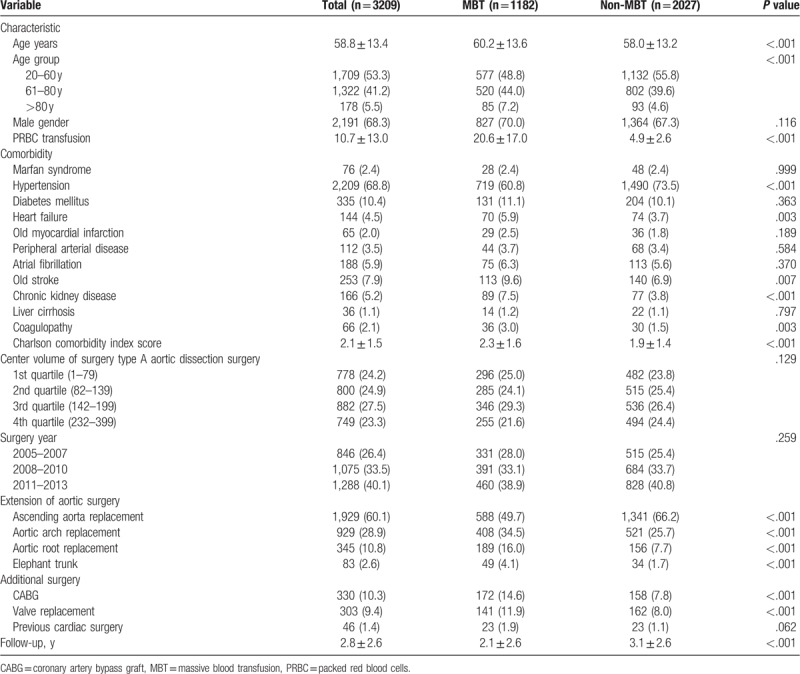
Clinical and surgical characteristics of study population.

## 3 Results

### 3.1 Study sample characteristics

Table 1 lists the baseline patient characteristics. A total of 3209 patients who underwent surgical repair of AAAD from 2005 to 2013 were included in this study (Fig. 1). Among these patients, 1182 individuals (36.8%) had an MBT ≥10-unit RBC during the perioperative period. These patients had an average age of 60.2 ± 13.6 years and were predominantly men (70.0%). Compared to the non-MBT group, patients with MBT tended to have a higher prevalence of coexisting medical conditions, such as heart failure, old stroke, CKD, and coagulopathy, and higher comorbidity index scores. Patients were more likely to receive MBT in those who underwent elephant trunk, followed by aortic root replacement, aortic arch replacement, and ascending aorta replacement. Patients with MBT were also more likely to receive additional surgeries, such as coronary artery bypass grafting and valve replacement. However, there was no significant difference between the MBT and non-MBT groups for history of any previous cardiac surgery.

**Figure 2:**
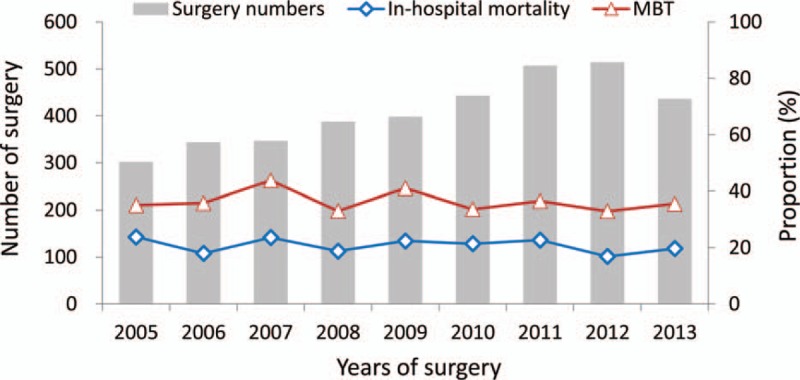
The epidemiology information of the number of surgical repair for type A aortic dissection, in-hospital mortality, and massive blood transfusion during the period from 2005 to 2013 in Taiwan.

Figure 2 shows the epidemiology of AAAD surgery in Taiwan from 2005 to 2013. The volume of AAAD surgeries increased gradually across the years (302 in 2005 and 436 in 2013). The proportion of patients receiving MBT and the rate of in-hospital mortality remained unchanged across the study years (for MBT, 35.1% in 2005 and 35.6% in 2013; for in-hospital mortality, 23.8% in 2005 and 19.7% in 2013).

### 3.2 Perioperative complications and in-hospital mortality

Results of surgery-related complications and outcomes are presented in Table 2. Patients with MBT during surgical repair of AAAD had a higher risk of adverse complications such as cardiogenic shock, respiratory failure, new onset ischemic/hemorrhagic stroke, re-exploration for bleeding, acute kidney injury with dialysis, sepsis, and in-hospital mortality. Patients receiving MBT were more likely to require blood products, including fresh frozen plasma and platelets. Furthermore, compared to non-MBT patients, patients with MBT had increased length of intensive care unit stay and hospital stay, and more medical expenditure.

**Table 2:**
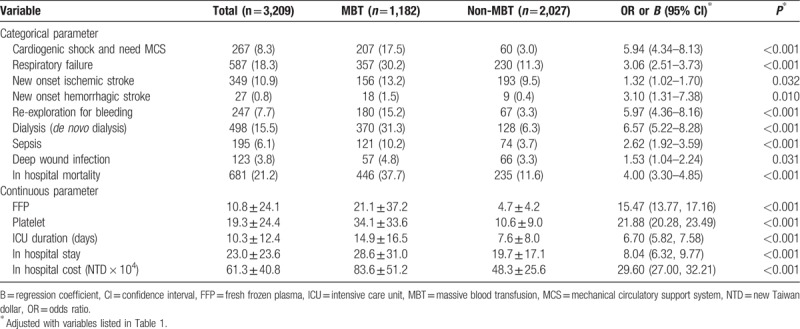
Operation-related complications and outcomes.

Table 3 shows the risk factor analysis for MBT after AAAD repair surgery. The significant covariates in the univariate analysis were introduced into a multivariate model with backward selection. The results indicated that the following variables were risk factors: older age, CKD, coagulopathy, extension of aortic surgery (including aortic arch replacement, arch root replacement, and elephant trunk), and additional surgery for coronary artery bypass grafting.

**Table 3:**
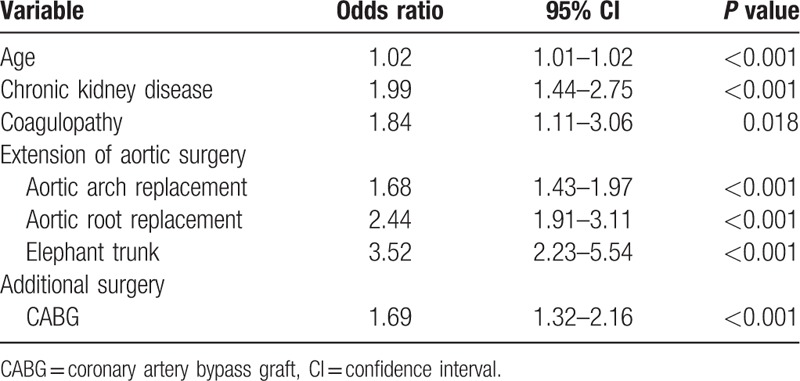
Risk factor analysis of massive blood transfusion after type A dissection repair.

### 3.3 Follow-up outcome

**Figure 3:**
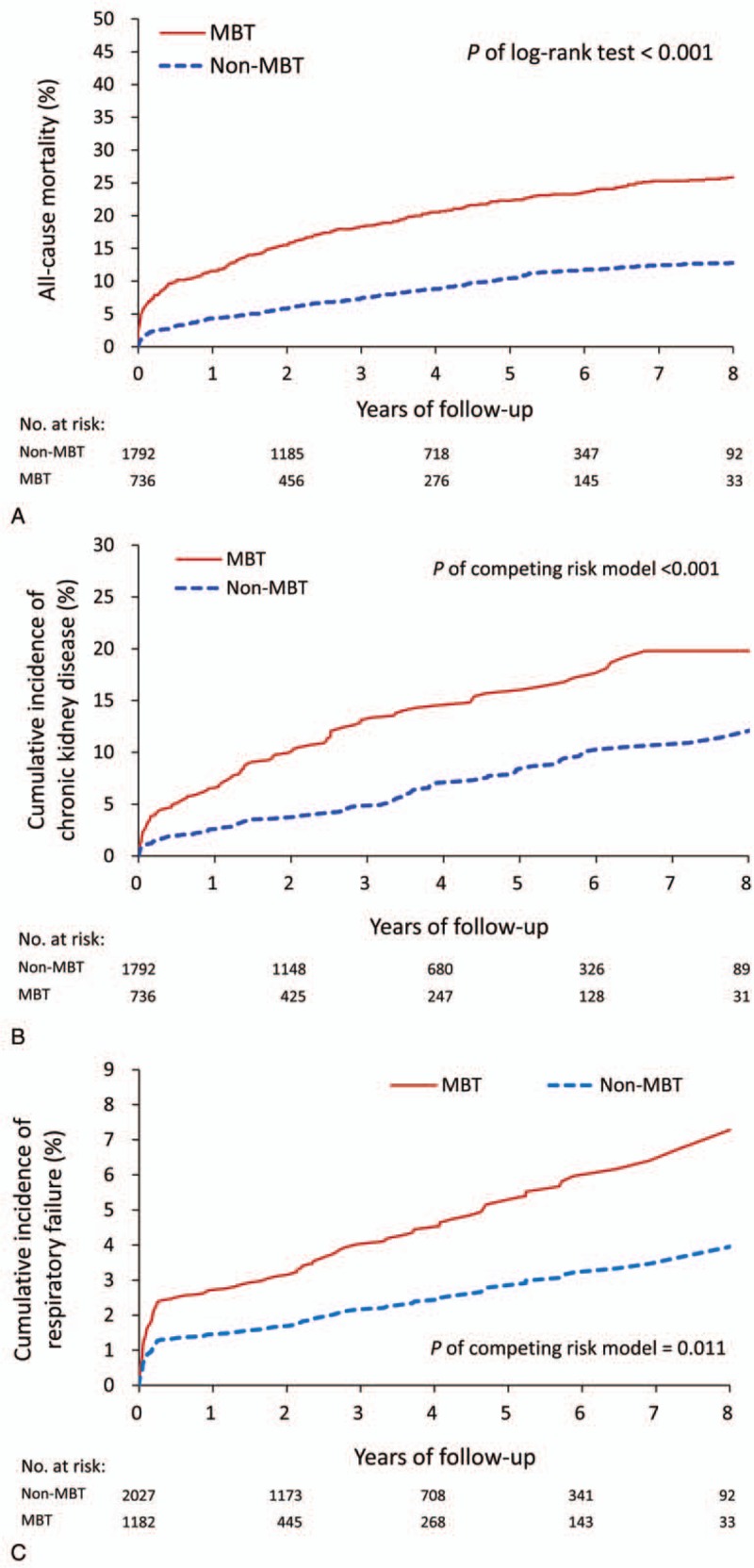
Survival curve estimated on the basis of multivariate Cox model for adjusted probability of all-cause mortality (A), cumulative incidence of chronic kidney disease (B), and cumulative incidence of respiratory failure (C) in patients who survived the index hospitalization during the study period.

**Figure 4:**
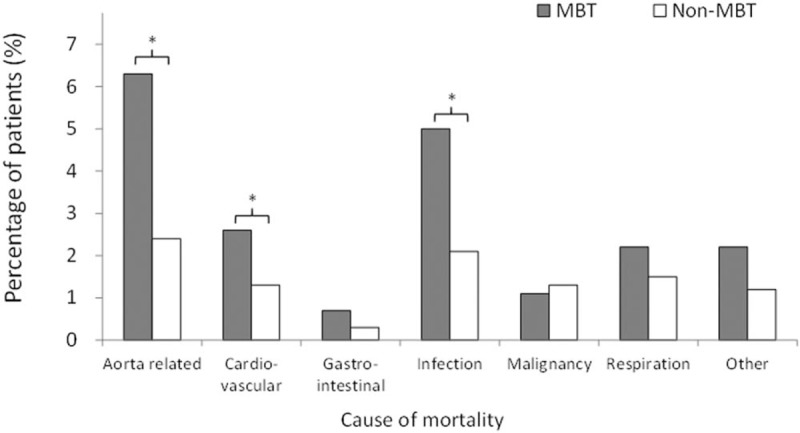
The subgroup analysis for causes of mortality. ^**∗**^*P* value < .05.

During the follow-up period, the MBT group had a higher mortality risk than the non-MBT group (54% vs 23%; hazard ratio [HR] 2.31; 95% confidence interval [CI] 2.04–2.62; data not shown). After excluding patients who died during the index hospitalization, the MBT group still demonstrated a significantly higher risk of mortality (26.1% vs 13.0%; HR 1.66; 95% CI 1.36–2.04; see Fig. 3A). Otherwise, Fig. 3B and C depict the cumulative incidence of CKD and respiratory failure after considering death as a competing risk. The results revealed that the MBT group had higher incidence of both CKD (13.9% vs 6.5%; HR 1.95; 95% CI 1.47–2.60) and respiratory failure (7.1% vs 2.7%; HR 2.34; 95% CI 1.52 –3.61) during the follow-up. Figure 4 shows that the subgroup analysis for causes of mortality. The proportions of aorta-related, cardiovascular-related, and infection-related death were observed in the MBT group.

**Figure 5:**
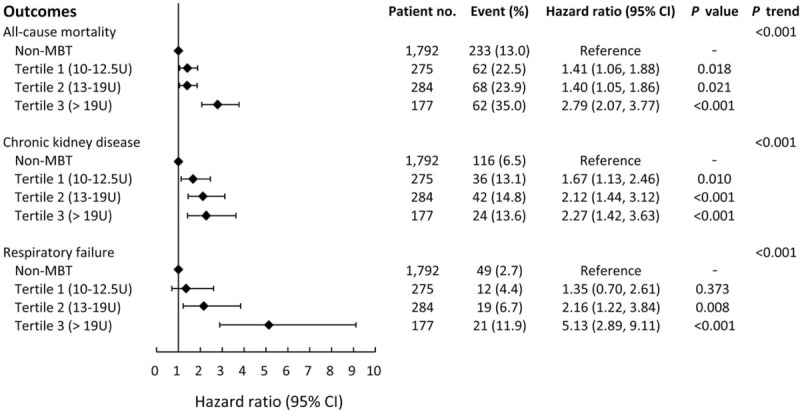
Relationship between the transfused volume of RBC and variables of late outcome (all-cause mortality, chronic kidney disease, and respiratory failure) in patients who survived the index hospitalization during the study period.

For MBT patients, the actual units of RBC used during surgical repair of AAAD were sorted into 3 group using tertile classification. Patients died during index hospitalization were excluded in this subgroup analysis. It appears that the greater the volume of RBC that was transfused, the higher the mortality rate after discharge. In the MBT 3rd tertile group (≥19 units), over one third (35.0%) of the patients died after index hospitalization. In addition, the volume of MBT was also associated with higher incidence of both CKD and respiratory failure after discharge (Fig. 5).

## 4 Discussion

To our knowledge, this article is the first retrospective, nationwide cohort study to investigate the impact of MBT on both short and late outcomes after surgical repair of AAAD.

The key findings of this study indicated higher incidence of in-hospital mortality (37.7% vs 11.6%, *P* < .001) following repair surgery in MBT group. After discharge, patient with MBT was tend to have higher all-cause mortality (26.1% vs 13%, *P* < .001), incidence of postoperative CKD (13.9% vs 6.5%, *P* < .001), and respiratory failure (7.1% vs 2.7%, *P* < .001) following repair surgery. A dose-dependent relationship was observed between the amount of transfused RBC (classified as tertiles) and both the cumulative incidence of mortality and respiratory failure (*P* trend <.001).

AAAD is acknowledged as a life-threatening condition, requiring emergent repair surgery, with high surgical and in-hospital mortality. In clinical practice, excessive bleeding related to repair of AAAD is a well-recognized problem.^[8]^ Consumptive coagulopathy due to blood flow through a false lumen, thrombocytopenia due to depletion of platelets at sites of collagen exposure, and hemodilution and increased platelet activation and aggregation owing to cardiopulmonary bypass, all lead to massive bleeding during aortic dissection and surgical repair of AAAD. Therefore, profuse bleeding is common in AAAD repair and MBT is consequently required.

MBTs are associated with adverse surgical outcomes, including operative death, surgical site infections, and pulmonary complications in trauma patients.^[17]^ However, reports on MBT in surgical repair of aortic dissection are lacking. In trauma patients, the mortality rate is as high as 40% with transfused RBC more than 10 units.^[18]^ These data are widely consistent with our findings. Several surgery-related complications, such as respiratory failure, renal failure, stroke, re-exploration for excessive bleeding, sepsis, and in-hospital mortality, were predominant in the MBT group following surgical repair of AAAD in our study.

Some studies have suggested that blood transfusion might induce pulmonary failure. The estimated incidence of transfusion-related acute lung injury as one form of acute lung injury is reported to be 1 in 8000 up to 1 in 5000 transfusions.^[19,20]^ In MBT patients, the rate of acute lung injury is higher, ranging from 24% to 36%.^[21]^ Furthermore, MBT might be associated with stroke and renal failure. The hypothesized basis for these complications is micro- and macrovascular thrombosis resulting from assumption coagulopathy in aortic dissection and MBT. Speculatively, the thrombotic status might lead to hypoperfusion, further elevated p-lactate levels, and higher rates of stroke and renal failure.^[22,23]^

Postoperative excessive bleeding requiring re-exploration surgery is more frequent in MBT patients.^[24]^ MBT can create serious hemostasis dysfunction, often caused by the combination of dilutional and consumptive effects of clotting factors and hyperfibrinolysis. Moreover, the impaired hemostatic condition can be aggravated in aortic dissection because of pathophysiological damage to the hemostatic system, cardiopulmonary bypass use, systemic hypothermia for brain protection, and complicated surgical technique.^[8–10,24]^ A higher incidence of reoperation for bleeding is also noted in our study.

A former study found that the degree of reduction in survival rates depended on the amount of RBC transfused in cardiac surgical patients.^[25]^ In our article, we divided actual units of transfused RBC into 3 groups using tertile classification. A statistically significant higher mortality rate was found in the tertile 3 group (>19 units RBC) compared to the tertile 1 and 2 groups (<19 units RBC) in a dose-dependent manner during follow-up period. In addition, a positive correlation was also observed with the cumulative occurrence of CKD and respiratory failure. Stored RBC has been documented to have limitations in oxygen delivery to organ tissues to depletion of 2,3 diphosphoglyceride, finally leading to damage of organs.^[26]^ Further, when the transfused volume of RBC is massive, it induces a large inflammatory load and enhances the inflammatory damage from cardiopulmonary bypass.^[27]^ The circulating component of blood cells alters after MBT, having implications for lymphocytes number, modification of T-cells, and activation of other immune cells. Those changes following MBT may be long-term influential.^[28]^

In sum, it is reasonable to assume that MBT in repair surgery for AAAD increases the incidence of adverse complications and the mortality rate. Therefore, clinicians should be cautious about the execution of MBT in cardiac surgical patients. In order to reduce the negative effects of MBT on postoperative outcome, there are numerous novel strategies involving alternative medications (recombinant activated factor VII/fibrinogen concentrate/tranexamic acid) and nearly real-time coagulation laboratory monitoring.^[9,29–31]^ However, investigation on long-term clinical testing of these new materials is still lacking. Therefore, further study and well controlled randomized trials are warranted in the future to achieve better postoperative outcomes.

### 4.1 Study strengths

This study possesses several strengths. First, to our knowledge, this is the first large-scale study with both short-term and late outcomes following MBT in cardiac surgery. Owing to the large representative sample size, the statistical power is adequate for analyzing the outcomes in this study. Second, the NHIRD contains complete, detailed information for the evaluation of outcomes of the in-hospital period and mid-term follow-up via the readmission records. This provides a powerful platform for detailing late outcomes. Finally, the NHIRD program covers almost all Taiwanese residents, and the screening examination before blood transfusion, as well as the actual transfused volume of any kind blood product, are all included via the declare provision system of NHIRD. Therefore, the declare mechanism for blood transfusion could substantially reduce the bias due to inaccurate information.

### 4.2 Limitations

There are several limitations that should be noted. First, specific details of the clinical patient's condition and imaging for diagnosis (computed tomography, magnetic resonance scanning, or echocardiography) are not available in the database. Aortic anatomy-specific data, such as aortic size, extent, or morphology present in preoperative images, and clinical vital status of patients, are the relatively leading factors affecting surgery difficulty, coagulaiton function, and postoperative outcome. For patients with refractory bleeding or requiring difficult surgical procedure, MBT is usually necessary for life support. Although image information is lacking, the image report is checked via the verifying system of the NHI Bureau to ensure medical consistency in our nation. This system ensures that bias is kept to a minimum.

Second, the NHIRD is a database for billing purpose, therefore biochemistry values like hemoglobin, hematocrit, platelet numbers, and coagulation tests were not available, which are crucial for the determination whether to initiate blood transfusion. Critical preoperative conditions (organ malperfusion and cardiac tamponade) also did not provide in NHIRD. Otherwise, intact information for during surgical process, such as surgical time, medication, vital sign of patients, laboratory data, and special procedure (like methods of brain protection), utilization of real-time tools for coagulation detection (Thrombelastography), and timing of blood transfusion, were not recorded in NHIRD. However, Taiwan's Bureau of NHI is responsible for auditing payments of laboratory tests, exams, medications, interventions, and surgeries, by comprehensive review of medical records. There is also a panel review system overseen by expert physicians to prevent abuse of blood transfusions by issuing fines for punishment. This auditing system could substantially mitigate the bias carried by overuse of blood transfusions.

Despite these limitations, the accuracy of NHIRD has been validated in patients with cardiovascular disease and cardiac surgery. NHIRD covered nearly 99% residents of Taiwan, and its reimbursement for medical treatment allowed it as a valid resource for population research. This way, we believe that our findings could provide a significant contribution to outcome analysis for patients with MBT in surgery for AAAD.

## 5 Conclusions

In this population-based nationwide study, patients with MBT in AAAD repair surgery had significantly poorer outcomes than those without MBT. Perioperative application of MBT was correlated with higher incidence of chronic renal failure, respiratory failure, and mortality during the follow-up period. Furthermore, a dose-dependent relationship was found between the amount of transfused RBC and both the cumulative incidence of mortality and respiratory failure. Recently, progress in novel surgical techniques and medication, or close coagulation surveillance, has successfully reduced the unnecessary volume of blood product in major surgeries. However, investigation on long-term clinical testing of these new materials is still lacking. Therefore, further study and well controlled randomized trials are warranted in the future to achieve better postoperative outcomes.

## Acknowledgments

The authors thank a grant from the Chang Gung Medical Research Project (BMRPC19, CMPRG3D1471, CMRPG3D1472, CMRPG3D1473, CORPG3G0591, CMRPG3G0601, CORPG3G0611, CORPG3G0621), Chang Gung Memorial Hospital, Linkou, Taiwan, and the Ministry of Science and Technology, Taiwan (MOST 106-2314-B-182A-061-MY2) for the support. Drs Shao-Wei Chen, An-Hsun Chou, Fang-Ting Chen, Victor Chien-Chia Wu, Chia-Hung Yang, Pao-Hsien Chu, and Pei-Chi Ting had full access to all the data used in the study and take responsibility for the integrity of the data and accuracy of analysis. The authors also thank Alfred Hsing-Fed Lin and Zoe Ya-Jhu Syu for their assistance in statistical analysis.

## Author contributions

**Conceptualization:** Fang-Ting Chen, An-Hsun Chou.

**Data curation:** Fang-Ting Chen, An-Hsun Chou, Victor Chien-Chia Wu, Chia-Hung Yang, Pao-Hsien Chu, Pei-Chi Ting.

**Formal analysis:** Fang-Ting Chen, An-Hsun Chou, Victor Chien-Chia Wu, Chia-Hung Yang, Pao-Hsien Chu, Pei-Chi Ting.

**Methodology:** Shao-Wei Chen.

**Supervision:** Shao-Wei Chen.

**Writing – original draft:** Fang-Ting Chen.

**Writing – review & editing:** Shao-Wei Chen.
